# In Vitro and In Ovo Evaluation of the Potential Hepatoprotective Effect of Metformin

**DOI:** 10.3390/medicina58060705

**Published:** 2022-05-25

**Authors:** Gabriel Veniamin Cozma, Alexandru Apostu, Ioana Macasoi, Cristina Adriana Dehelean, Octavian Marius Cretu, Stefania Dinu, Dan Gaiță, Aniko Manea

**Affiliations:** 1 Department of Surgical Semiology, Faculty of Medicine, “Victor Babeş” University of Medicine and Pharmacy Timisoara, Eftimie Murgu Square No. 2, 300041 Timişoara, Romania; drgabrielcozma@gmail.com; 2 Department of Cardiology, “Victor Babeş” University of Medicine and Pharmacy Timisoara, 49 No., C. D. Loga Bv., 300041 Timişoara, Romania; dr.apostualex@gmail.com (A.A.); dgaita@cardiologie.ro (D.G.); 3 Advanced Research Center of the Institute for Cardiovascular Diseases, “Victor Babeş” University of Medicine and Pharmacy Timisoara, Eftimie Murgu Square No. 2, 300041 Timişoara, Romania; 4 Departament of Toxicology and Drug Industry, Faculty of Pharmacy, “Victor Babeş” University of Medicine and Pharmacy Timisoara, Eftimie Murgu Square No. 2, 300041 Timişoara, Romania; 5 Research Center for Pharmaco-Toxicological Evaluations, Faculty of Pharmacy, “Victor Babeş” University of Medicine and Pharmacy Timisoara, Eftimie Murgu Square No. 2, 300041 Timişoara, Romania; 6 Department of Surgery, Faculty of Medicine, “Victor Babeş” University of Medicine and Pharmacy Timisoara, Eftimie Murgu Square No. 2, 300041 Timişoara, Romania; tavi@octaviancretu.ro; 7 Department of Pedodontics, Faculty of Dental Medicine, “Victor Babeş” University of Medicine and Pharmacy Timisoara, 9 No., Revolutiei Bv., 300041 Timişoara, Romania; dinu.stefania@umft.ro; 8 Pediatric Dentistry Research Center, Faculty of Dental Medicine, “Victor Babeş” University of Medicine and Pharmacy Timisoara, 9 No., Revolutiei Bv., 300041 Timişoara, Romania; 9 Department of Obstetrics and Gynecology, Faculty of Medicine, “Victor Babeş” University of Medicine and Pharmacy Timisoara, Eftimie Murgu Square No. 2, 300041 Timişoara, Romania; aniko180798@yahoo.com

**Keywords:** metformin, hepatoprotective, anti-irritant, chorioallantoic membrane, HET-CAM

## Abstract

*Background and Objectives*: Metformin is currently the leading drug of choice for treating type 2 diabetes mellitus, being one of the most widely used drugs worldwide. The beneficial effects of Metformin, however, extend far beyond the reduction of blood glucose. Therefore, this study aimed to evaluate Metformin’s effects both in vitro and in ovo. *Materials and Methods:* Metformin has been tested in five different concentrations in human hepatocytes —HepaRG, in terms of cell viability, morphology, structure and number of nuclei and mitochondria, as well as the effect on cell migration. Through the application of HET-CAM, the biocompatibility and potential anti-irritant, as well as protective effects on the vascular plexus were also assessed. *Results:* According to the results obtained, Metformin increases cell viability without causing morphological changes to cells, mitochondria, or nuclei. Metformin displayed an anti-irritant activity rather than causing irritation at the level of the vascular plexus. *Conclusions:* In conclusion, Metformin enhances cell viability and proliferation and, has a protective effect on the vascular plexus. Nonetheless, more studies are required to clarify the mechanism of hepatoprotective effect of metformin.

## 1. Introduction

Type 2 diabetes mellitus is characterized by hyperglycemia and dyslipidemia as a result of the inability of islet β-cells to secrete insulin. This pathology has become a burden for modern society due to the explosion of the number of cases worldwide and multi-organ damage [1]. Clinical observations have shown that the low life expectancy of diabetic patients is not only related to vascular complications and kidney disease, but also to liver damage. The most common liver complications associated with diabetes are liver cirrhosis and hepatocellular carcinoma [2].

Metformin (Met) was introduced as a therapy in 1958 in the United Kingdom and has since become the most widely used drug for the treatment of type 2 diabetes. Met lowers blood glucose levels by decreasing plasma insulin levels and increasing insulin sensitivity and peripheral glucose uptake. In addition to these mechanisms of action, Met is involved in the process of gluconeogenesis by activating AMP protein kinase (AMPK) [3].

The use of Met goes beyond its primary indication, as it is also used for other pathologies, such as obesity, metabolic syndrome, or polycystic ovary syndrome [4]. Since Met has been used in therapy for over 50 years, alternative potential therapeutic activities are currently being investigated and intensively discussed, such as its antitumoral [5], anti-aging action [6], or cardioprotective action [7]. Particular attention was paid to the action of Met on the liver and striated muscles as a result of activating AMPK and inhibiting the mitochondrial complex 1. Inhibition of mitochondrial complex 1 is the mechanism by which Met mediates its effects through AMPK, thereby decreasing reactive oxygen species in the complex [8].

Met is attracting researchers’ attention because of the possibility that it may have antitumor activity as demonstrated in a retrospective report, indicating enhanced survival rates in cancer patients treated with Met [4]. Met treatment has been associated with the prevention of hepatocellular carcinoma and a decrease in the number of deaths associated with it [9]. These findings are the result of a clinical study that showed a more than 80% decrease in the chances of developing hepatocellular carcinoma in cirrhotic patients treated with Met compared to patients receiving exogenous insulin or secretagogin insulin [10].

Met reduces serum glucose levels by inhibiting hepatic gluconeogenesis and glycogenolysis in addition to enhancing striated muscle glucose uptake, inhibiting fatty acid oxidation, and reducing triglyceride levels. All these actions of Met cause a beneficial effect on the liver by reducing steatosis and improving liver enzymes [10,11]. The mechanisms underlying the protective effect of Met can be classified into direct mechanisms and indirect mechanisms. A decrease in plasma insulin levels is directly responsible for the hepatoprotective effect. On the other hand, the indirect mechanisms of preventing carcinogenesis are the induction of cellular apoptosis, the stimulation of the immune system and the activation of AMPK [12]. One of the consequences of diabetes is non-alcoholic fatty liver disease (NAFLD), which is considered a risk factor for hepatocellular carcinoma. Met has a protective effect against NAFLD by increasing liver enzymes and reducing obesity and steatosis [13].

Moreover, drug-induced liver toxicity is also a major health concern. In many cases, the hepatotoxic potential is not detected during preclinical studies, due to the fact that hepatotoxicity may be idiosyncratic [14]. Among the best known substances with hepatotoxic effects are acetaminophen and ethanol [15]. There are studies that show the role of Met in the prevention of liver damage associated with the consumption of toxic substances [16,17,18].

The aim of the present study was to evaluate the in vitro and in ovo effect of Met using five concentrations (0.5, 1, 1.5, 2 and 2.5 mM). In in vitro studies, the effect exerted by Met on the hepatocyte cell line—HepaRG, was evaluated in terms of viability, cell morphology, influence on structures and number of nuclei and mitochondria and cell migration capacity. In ovo, the biocompatibility and irritant and anti-irritant potential of Met were evaluated by employing the chick chorioallantoic membrane (HET-CAM) assay.

## 2. Materials and Methods

### 2.1. Reagents

Metformin hydrochloride, trypsin-EDTA solution, phosphate saline buffer (PBS), dimethyl sulfoxide (DMSO), fetal calf serum (FCS), penicillin/streptomycin, insulin from bovine pancreas, Hydrocortisone 21-hemisuccinate sodium and MTT [3-(4,5-dimethylthiazol-2-yl)-2,5-diphenyltetrazolium bromide] reagents were purchased from Sigma Aldrich, Merck KgaA (Darmstadt, Germany). The cell culture media, William’s E Medium (Gibco™; 12551032) were purchased from Gibco, Waltham, MA, USA). All reagents were analytically pure and proper for cell culture use.

### 2.2. Cell Culture

The effect of Met on hepatocytes was studied in the HepaRG cell line provided by Gibco as frozen vials—HepaRG ™ Cells (Catalog number: HPRGC10). The cell line was cultured in a culture-specific medium, represented by William’s E Medium containing 4 μg/mL Insulin from the bovine pancreas, 5 μM Hydrocortisone 21-hemisuccinate sodium, 10% FCS and 1% penicillin (100 U/mL)-streptomycin (100 µg/mL) mixture. During the experiment, the cells were maintained at a standard temperature (37 °C) and 5% CO_2_.

### 2.3. Cellular Viability Assessment

The MTT method was used to determine cell viability. For this purpose, the cells were cultured in 96-well plates in a number of 1 × 10^4^ cells / well. After reaching a confluence of approximately 90%, the cells were stimulated with five different concentrations of Met (0.5, 1, 1.5, 2 and 2.5 mM) for two time intervals of 24 h and 72 h. Test concentrations of Met hydrochloride were prepared in the culture medium. After these time intervals, the stimulation medium was removed and replaced with fresh medium (100 μL/well); 10 μL of reagent MTT was added to each well, and the plates were placed in an incubator at 37 °C for 3 h. After this time, the solubilization solution was added to a volume of 100 μL/well, and the plates were incubated for 30 min at room temperature, away from light. Subsequently, the measurement of absorbents at two wavelengths of 570 and 630 nM was performed using Cytation 5 (BioTek Instruments Inc., Winooski, VT, USA).

### 2.4. Cellular Morphology

In order to determine the possible effects induced by Met regarding the morphology and confluence of HepaRG cells, a microscopic evaluation was performed by photographing the cells under bright field illumination after the time intervals of 24 h and 72 h. The pictures were analyzed using the Gen5 ™ Microplate Data Collection and Analysis Software (BioTek Instruments Inc., Winooski, VT, USA).

### 2.5. Immunofluorescence

For the immunofluorescence visualization of the cellular components, HepaRG was cultured in plates of 12 wells, with 1 × 10^5^ cells / well, and after reaching a corresponding confluence of approximately 90%, they were stimulated for 72 h with Met (0.5, 1, 1.5, 2 and 2.5 mM). After 24 h, the cells were washed with ice-cold PBS and then fixed with paraformaldehyde 4% by maintaining room temperature for one hour. Cell permeabilization was performed with Triton X 2% in PBS. Then, 30% FBS solution in 0.01% Triton X was used for one hour to block Triton X 2% action. The cells were incubated at 4 °C overnight with Anti-COX IV antibody Mitochondrial marker (ab33985) at a dilution. of 1:500 for mitochondrial visualization. The next day, the primary antibody was washed with 0.01% Triton X solution in PBS and the secondary antibody specific for COX IV mitochondrial marker—Donkey Anti-Goat IgG H&L (Alexa Fluor^®^ 488—ab150129) was added, and the plate was kept for 2 h at room temperature and protected from light. After this time, 4′, 6-diamidino-2-phenylindole (DAPI) staining was added to visualize the nuclei. The pictures were then taken with Cytation 1 and processed using Gen5 ™ Micro-plate Data Collection and Analysis Software (BioTek^®^ Instruments Inc., Winooski, VT, USA).

### 2.6. Wound Healing Assay

The wound healing (scratch) test was used to assess the effect of Met on cell migration. For this purpose, the cells were cultured in 24-well Corning plates (1 × 10^5^ cells/ well) and an automatic scratch was made in the middle of each well using the AutoScratch ™ Wound Making Tool provided by BioTek^®^ Instruments Inc., Winooski, VT, USA as recommended by the manufacturer. Cells were then stimulated with the five Met concentrations (0.5, 1, 1.5, 2 and 2.5 mM) for a 24-hour interval. Photographs (4 × magnification) were taken both at the beginning of the experiment (0 h) and after the stimulation was completed (24 h) using Cytation 1 and were processed using Gen5 ™ Microplate Data Collection and Analysis Software (BioTek^®^ Instruments Inc., Winooski, VT, USA). The effect of Met on cell migration was quantified by calculating the migration rate, expressed as a percentage, using the formula described above [19].

### 2.7. Chorioallantoic Membrane (CAM) Assay

Chicken eggs (*Gallus gallus domesticus*) obtained from a local farmer were used to evaluate the effect of Met on the vascular plexus. They were disinfected with 70% alcohol (*v*/*v*), and after noting the date on them, they were placed in the incubator, under standard conditions of temperature (37 °C) and humidity in a horizontal position. The eggs were then prepared for the experiment as follows: (i) on the fourth day of incubation, a perforation was made at the level of the eggshell through which a volume of 7 mL of albumen was extracted in order to favor the detachment of the membrane from the inner shell of the egg; (ii) on the fifth day, a window was cut at the upper level of the egg, in order to allow the visualization of the chorioallantoic membrane. The cut-out window was then covered with duct tape, and eggs were placed in the incubator until the day the experiment began.

### 2.8. Hen’s Egg Test—Chorioallantoic Membrane (HET-CAM) Assay

The Hen’s Egg Test (HET-CAM) method was applied to assess biocompatibility and possible irritant potential at the vascular level. The experiment was performed on the ninth day of incubation. For this purpose, Met was tested at 2 mM, at which the most beneficial in vitro effects were previously observed. In parallel, a negative control—H_2_O and a positive control—1% sodium dodecylsulfate (SDS) were used. Both the test sample and the two controls were applied to the chorioallantoic membrane at a volume of 600 μL so that the entire surface of the membrane was covered. The changes observed at the vascular level were vascular lysis (*L*), coagulation (*C*) and hemorrhage (*H*). These changes were observed for a period of 5 min after application to the samples, using a stereomicroscope (Discovery 8 Stereomicroscope, Zeiss, Göttingen, Germany) and the images were taken (Axio CAM 105 color, Zeiss) 5 min before and after application to the samples. To quantify the potential irritant effect, the irritation score (*IS*) was calculated using the formula [20]:

$$IS=5\times \frac{301-H}{300}+7\times \frac{301-L}{300}+9\times \frac{301-C}{300}$$

where *H* represents hemorrhage, *L* represents vascular lysis, and *C* represents intravascular coagulation.

Depending on the value of the irritation score, the substances can be classified into three categories, as follows: (i) Non-irritating (*IS* = 0–0.9); (ii) Irritating (*IS* = 1–8.9) and (iii) Severe irritating (*IS* = 9–21) [21].

### 2.9. Determination of Anti-Irritative Potential

To determine the potential anti-irritant effect, the eggs were prepared as previously described, and the method applied was the one described above [22] and adapted to our laboratory conditions. Thus, to determine the anti-irritant potential, Met was tested at a concentration of 2 mM, previously tested in the HET-CAM assay, together with the negative control represented by H_2_O. The samples were applied to the chorioallantoic membrane at a volume of 600 μL, and then the eggs were placed in the incubator for a period of 3 h so that the solutions were absorbed into the membrane. After this time, the eggs were treated with 300 μL 1% sodium dodecyl sulfate (SDS), and the vascular plexus was examined for changes similar to those previously observed in the HET-CAM method (lysis, stasis, and hemorrhaging). The changes were observed for a period of 5 min, with photographs being taken before and after application to the samples. The same parameters from the HET-CAM test (*H*, *L* and *C*) were followed, for calculating the following parameters:

$H_{AI\;}=\frac{H}{H_{SDS}}\;$; (hemorrhage time after pretreatment with Met 2 mM and 1% SDS addition/hemorrhage time without pretreatment with Met 2 mM);

$L_{AI\;}=\frac{L}{L_{SDS}}\;$; (vascular lysis time after pretreatment with Met 2 mM and 1% SDS addition/vascular lysis time without pretreatment with Met 2 mM);

$C_{AI\;}=\frac{C}{C_{SDS}}\;$; (vascular coagulation time after pretreatment with Met 2 mM and 1% SDS addition/vascular coagulation time without pretreatment with Met 2 mM).

### 2.10. Statistical Analysis

All data are expressed as means ± SD, the differences being compared by applying the one-way ANOVA analysis followed by Dunnett’s multiple comparisons post-test. The used software was GraphPad Prism version 9.0.0 for Windows (GraphPad Software, San Diego, CA, USA, www.graphpad.com, accessed on 27 April 2022). The statistically significant differences between data were labeled with * (* *p* < 0.1; ** *p* < 0.01; *** *p* < 0.001; **** *p* < 0.0001).

## 3. Results

### 3.1. Cellular Viability Assessment

To determine the effect of metformin on hepatocytes, the viability of HepaRG cells was determined at 24 h and 72 h intervals after stimulation with Met at a concentration of 0.5, 1, 1.5, 2 and 2.5 mM.

**Figure 1:**
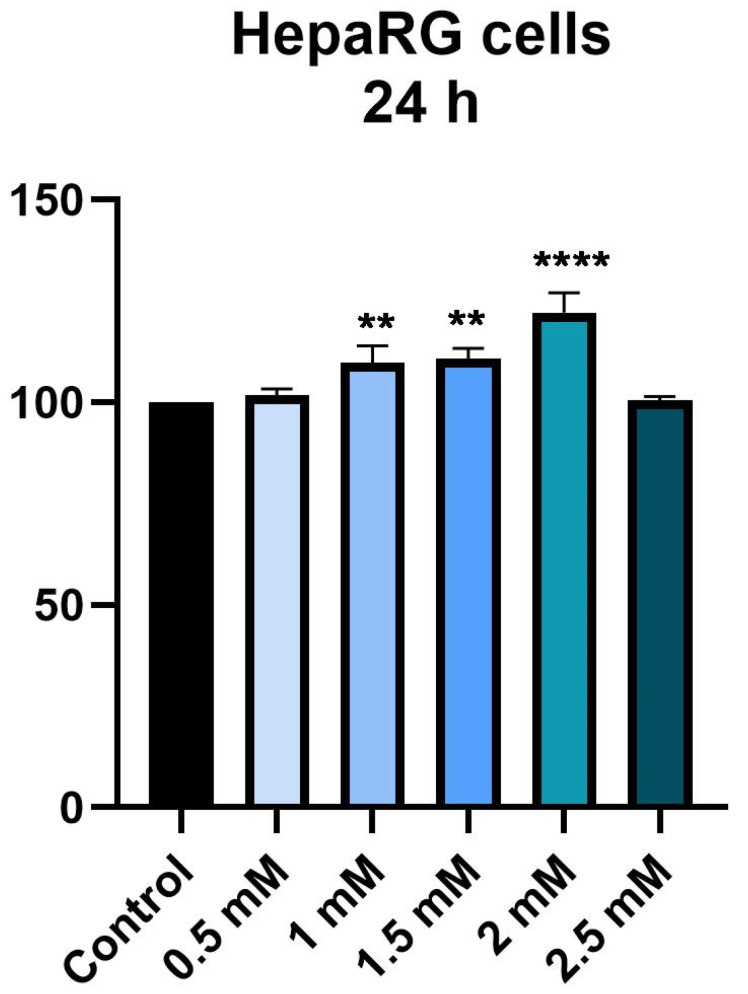
In vitro assessment of the effect of Met (0.5, 1, 1.5, 2 and 2.5 mM) exerts on the viability of HepaRG after 24 h of treatment. The data are expressed as viability percentages (%) normalized to control cells and expressed as mean values ± SD of three independent experiments performed in triplicate. To identify the statistical differences between the Control and the Met-treated group, a one-way ANOVA analysis was conducted followed by the Dunnett’s multiple comparisons post-test (** *p* < 0.01; **** *p* < 0.0001).

After 24 h, a concentration-dependent increase in cell viability was detected, up to 2 mM, after which the value of cell viability did not change compared to the control group. Thus, at the first two tested concentrations (0.5 and 1 mM) a slight increase in viability can be observed, of approximately 101% and 109%, respectively. The highest value of cell viability was recorded at a concentration of 2 mM (approximately 122%). As a result of the maximum concentration tested, 2.5 mM, a decrease in cell viability can be observed compared to the other concentrations tested, but not less than the control concentration (Figure 1). Thus, Met, at a time interval of 24 h, does not cause a decrease in cell viability, on the contrary, at a concentration of 2 mM it causes a significant increase.

**Figure 2:**
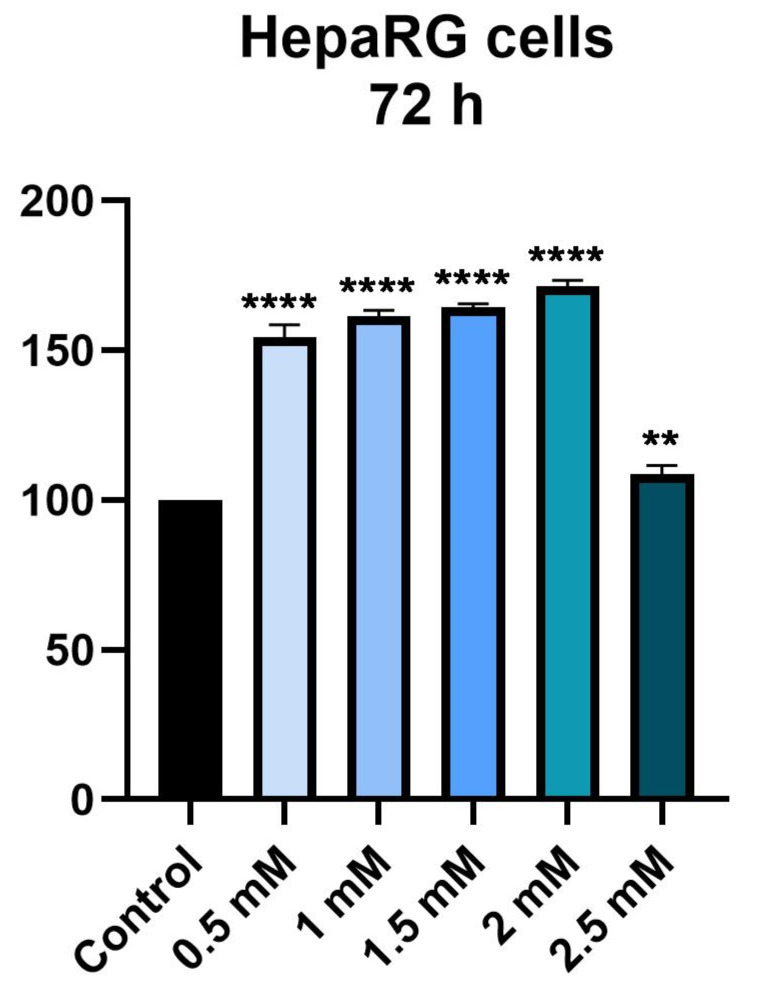
In vitro assessment of the effect of Met (0.5, 1, 1.5, 2 and 2.5 mM) exerts on the viability of HepaRG after 72 h of treatment. The data are expressed as viability percentages (%) normalized to control cells and expressed as mean values ± SD of three independent experiments performed in triplicate. To identify the statistical differences between the Control and the Met-treated group, a one-way ANOVA analysis was conducted followed by the Dunnett’s multiple comparisons post-test (** *p* < 0.01; **** *p* < 0.0001).

At the 72 h time interval, the same trend is observed in terms of influencing cell viability. Thus, at the first four concentrations tested (0.5, 1, 1.5 and 2 mM) there is an increase in cell viability depending on the concentration, while at a concentration of 2.5 mM there is a decrease in cell viability compared to the first concentrations tested; however, the value of cell viability was higher than that of the control group, with the viability being approximately 100% in this case. However, the highest value of cell viability was recorded at a concentration of 2 mM, being about 171% (Figure 2).

### 3.2. Cellular Morphology

The effect of Met on the morphology and confluence of HepaRG cells was evaluated and monitored by photographing the cells after two time intervals, 24 h and 72 h.

**Figure 3:**
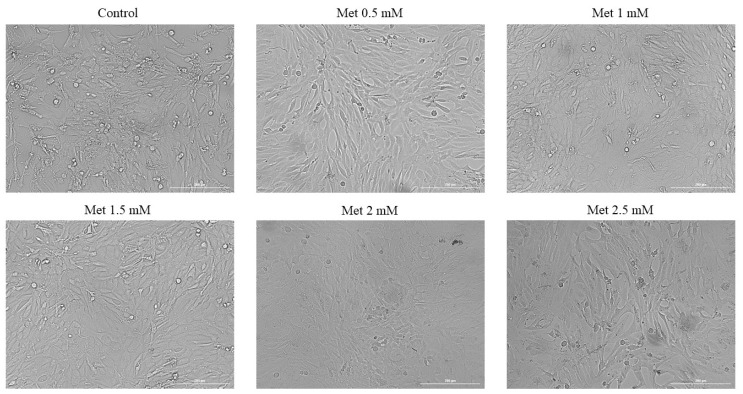
Morphology and confluence of HepaRG cells following the 24 h treatment with Met 0.5, 1, 1.5, 2 and 2.5 mM. The scale bars indicate 200 µm.

In addition to the results obtained from the previously conducted cell viability test, the morphology of the cells confirms the results obtained after stimulation for 24 h. In Figure 3, it can be observed that, for the first four tested concentrations, there is an increase in the number of cells and cell confluence. At a concentration of 2 mM, there was an increase in the percentage of cells confluent compared to the control; however, at the highest concentration, 2.5 mM, a slight decrease in the percentage of cells was observed, but the cell confluency was similar to that observed in the control.

**Figure 4:**
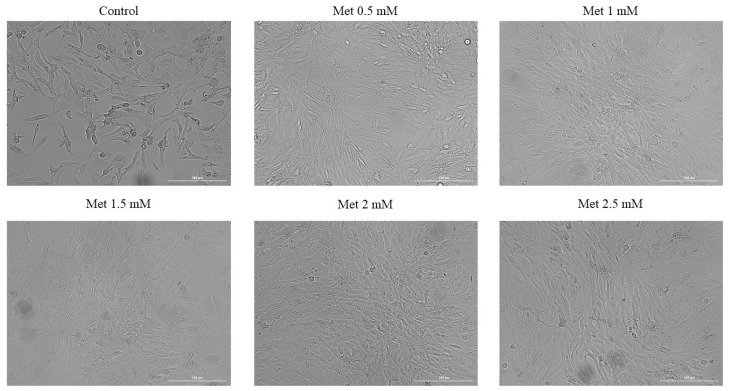
Morphology and confluence of HepaRG cells following the 72 h treatment with Met 0.5, 1, 1.5, 2 and 2.5 mM. The scale bars indicate 200 µm.

Regarding the time interval of 72 h, the results obtained are similar to those observed in the case of cell viability testing. Thus, at the first four concentrations tested, a dose-dependent increase in confluence and number of cells was observed, while at the highest concentration tested, 2.5 mM, there was a slight decrease in cell confluence. However, no change in cell morphology was observed in any of the cases, with the cells having morphological characteristics unaffected by Met treatment (Figure 4).

### 3.3. Immunofluorescence

**Figure 5:**
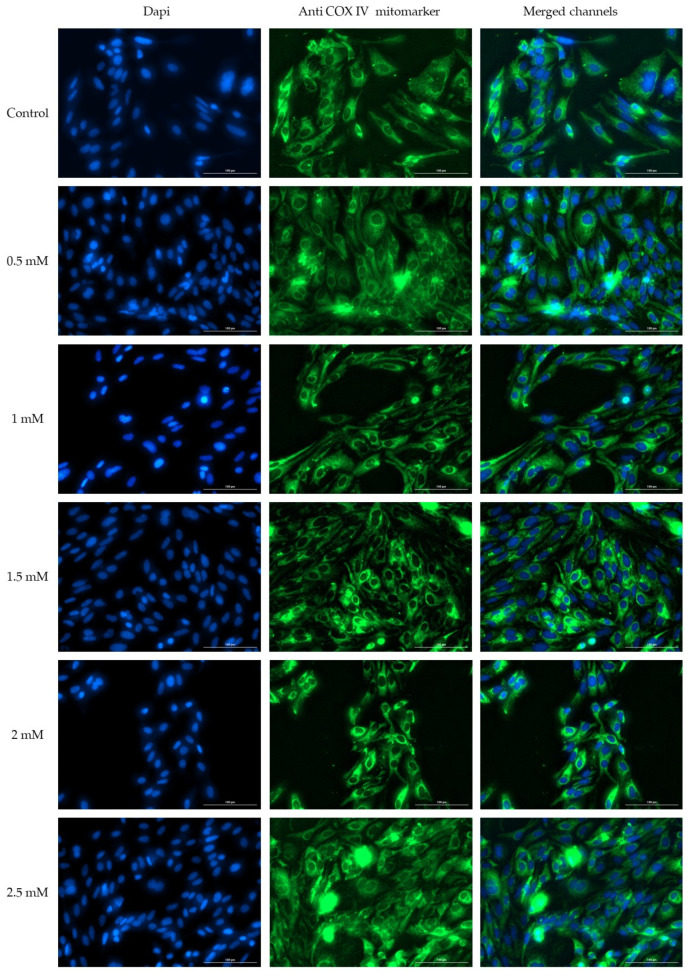
Effect of Met (0.5, 1, 1.5, 2 and 2.5 mM) after 72 h of stimulation on the HepaRG cells on nuclei—DAPI staining (blue) and COX IV mitochondrial marker (green).

To assess whether Met may exert effects on the morphology of the nucleus and mitochondria, the immunofluorescence method was applied. The tested concentrations showed no effect on cell morphology. Even in the case of the highest concentration tested, no significant changes were observed, such as condensation or a decrease in number. Similarly, in the case of mitochondria, treatment with Met does not cause changes in distribution and morphology. Thus, the mitochondria, in the case of the first four tested concentrations, show a uniform distribution, without signs of condensation or decrease in number. Instead, in the case of the highest concentration tested, a slight condensation of mitochondria around the nuclei is observed (Figure 5).

### 3.4. Wound Healing Assay

**Figure 6:**
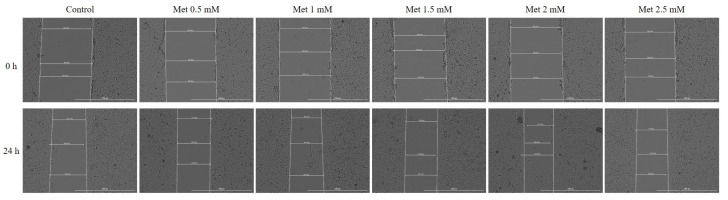
Representative images of the migratory capacity of HepaRG cells following the treatment with Met (0.5, 1, 1.5, 2 and 2.5 mM) for 24 h. Images were captured at 0 h and 24 h after stimulation with Met versus control cells. The scale bars indicate 1000 µm.

**Figure 7:**
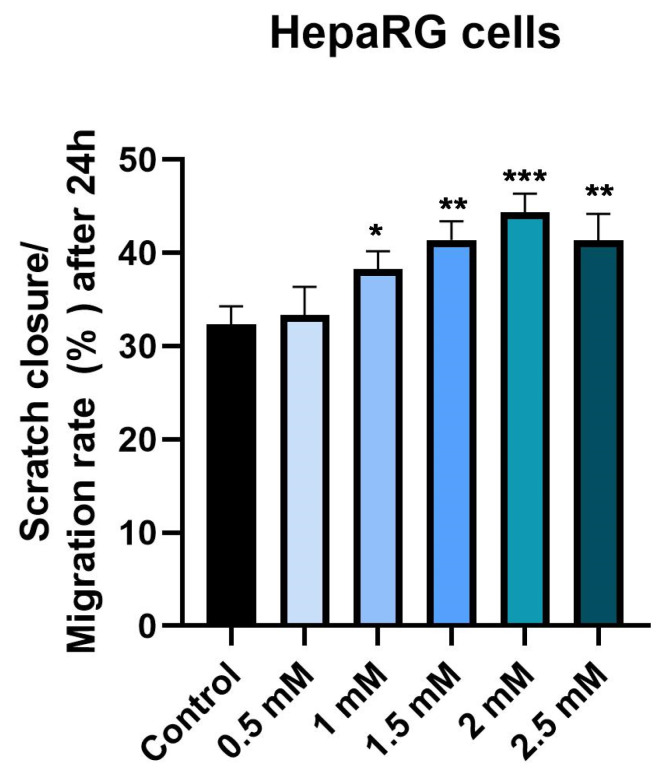
Scratch closure rate of HepaRG following Met treatment (0.5, 1, 1.5, 2 and 2.5 mM). The bar graphs are presented as wound closure percentage after 24 h compared to the initial surface. The data are expressed as mean values ± SD of three independent experiments performed in triplicate. The statistical differences between the Control and the Met-treated group were identified by applying the one-way ANOVA analysis followed by the Dunnett’s multiple comparisons post-test (* *p* < 0.05; ** *p* < 0.01; *** *p* < 0.001).

Considering that Met may have a liver repair effect and may interfere with hepatocyte regeneration, the potential effect on cell migration was investigated by the Wound Healing method. For this purpose, the five previously tested concentrations (0.5, 1, 1.5, 2 and 2.5 mM) were tested. The effects observed in this case support the results obtained previously. There is an increase in the concentration-dependent cell migration rate in the case of the first four concentrations, with the highest approach rate being recorded in the case of the 2 mM concentration, followed by a slight decrease in cell migration in the case of the 2.5 mM concentration (Figure 6). Figure 7 shows graphically the rate of cell migration depending on the metformin concentration tested.

### 3.5. Hen’s Egg Test—Chorioallantoic Membrane (HET-CAM) Assay

To determine the biocompatibility and evaluation of the irritant and toxic potential of Met in the vascular plexus, the HET-CAM method was applied, which is based on the calculation of the irritation score (*IS*). For the best evaluation, two types of controls were used, a negative control (H_2_0) and a positive control (SDS 1%), in parallel with testing the 2 mM Met solution.

**Figure 8:**
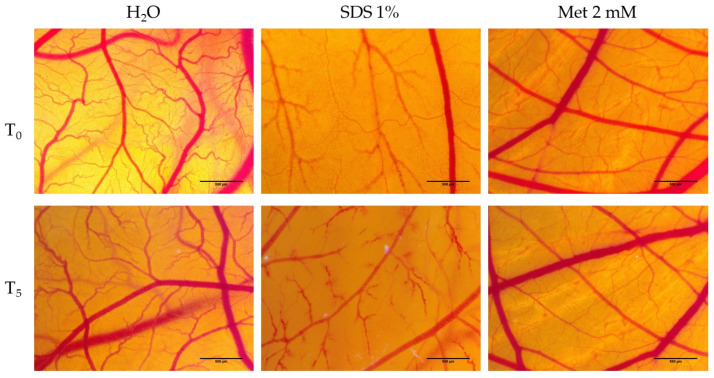
Analysis of the irritant potential of Met at concentration of 2 mM by the HET-CAM method. Stereomicroscopic images of CAMs inoculated with negative control—H_2_O, positive control—SDS and test compound—Met 2 mM.

Table 1 shows the irritation scores obtained for both the two controls and the Met solution. Not surprisingly, the highest irritation score was obtained in the case of the positive control (19.68). Figure 8 shows the irritating effects of SDS at the level of the vascular plexus, with noticeable hemorrhaging, lysis and vascular coagulation. Conversely, there is the negative control, represented by water, where the irritation score achieved was 0.13. Met did not produce significant irritancy effects, as indicated by the irritation score obtained in this case (0.7). Concerning the effects at the vascular level, there was only the appearance of slight intravascular coagulation (Figure 8). However, the viability of Met-treated specimens was good, and they were viable even after 24 h of application. In SDS, on the other hand, the death of the specimens was recorded less than one hour after application.

### 3.6. Determination of Anti-Irritative Potential

Based on the fact that Met does not cause vascular irritation, and that cell viability is stimulated after Met treatment, it was decided to assess Met’s protective and anti-irritant effects. Thus, pretreatment with Met and H_2_O was performed, followed by irritation with 1% SDS.

**Figure 9:**
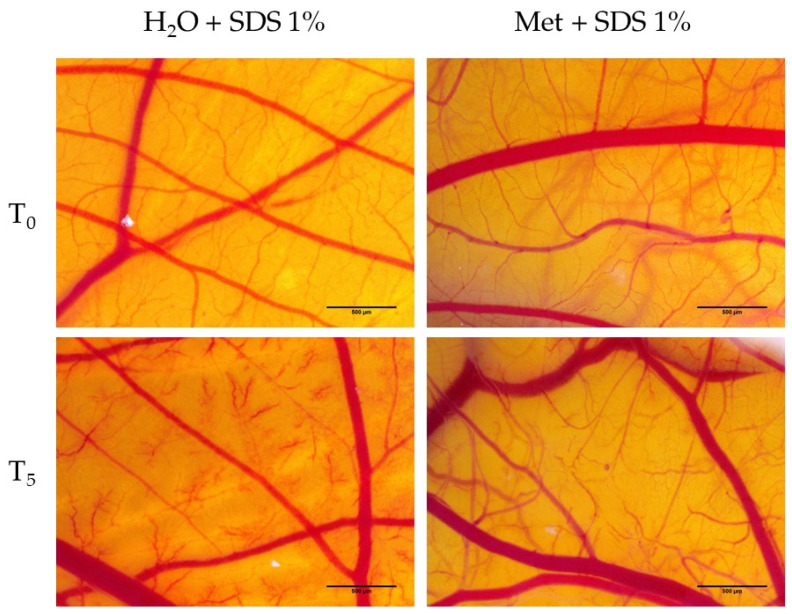
Stereomicroscopic images of CAM after pretreatment with Met 2 mM and H_2_O followed by 1% SDS irritation.

Table 2 shows the irritation scores obtained from pretreatment with H_2_O and Met. In the case of water, it was observed that the irritation score is not very different from that obtained by SDS 1% without pretreatment. In Figure 9, it can be seen that pretreatment of H_2_O does not prevent the occurrence of the vascular irritant effects exerted by SDS, as it is seen that both areas with vascular lysis and areas with hemorrhaging are present. On the other hand, the pre-treatment with Met 2 mM exerts a protective and anti-irritant effect, with the irritation score obtained in this case (7.29) being significantly lower than that obtained in the case of applying SDS without pretreatment. Within five minutes, the presence of small irritating effects at the vascular level was evident, manifested as slight vascular lysis, followed by intravascular coagulation and small areas of microhemorrhage (Figure 9).

## 4. Discussion

Met belongs to the class of biguanides and was introduced in the 1950s for the treatment of type 2 diabetes mellitus (T2DM). However, many other therapeutic effects of Met have been observed over time, and these are not limited to the treatment of diabetes mellitus. Thus, clinical trials have suggested the usefulness of Met in the treatment of polycystic ovary syndrome, obesity, cardiovascular complications and, most importantly, in reducing the risk and mortality of cancer, especially pancreatic, liver and lung cancer [15]. In addition, several research directions have shown the protective role of Met against liver damage by toxic substances, such as acetaminophen [23] or arsenic trioxide [16], as well as in the prevention of certain liver pathologies, such as liver cirrhosis [24].

The association between T2DM and liver damage has been recognized for over 30 years [25]. In patients with T2DM, liver damage can range from fatty liver disease/metabolic associated fatty liver disease, non-alcoholic steatohepatitis, and hepatocellular carcinoma to liver failure [26]. In addition to T2DM, T1DM can lead to liver complications, such as glycogenic liver disease and diabetic hepatosclerosis, recently described as a non-cirrhotic form of hepatic microangiopathy [27,28]. In addition to these two pathologies with known implications in the physiopathology of hepatotoxicity, a new clinical feature, hepatocellular diabetes, has recently been described. This condition can be described as a glucose disorder caused by impaired liver function [29].

Based on the premise that metformin may have a hepatoprotective effect, the present study aimed to evaluate the effect induced by Met on the hepatocyte cell line, HepaRG, in terms of viability, morphology and cell structures, such as nucleus and mitochondria. In addition, the effect of Met on the vascular plexus of the chorioallantoic membrane was evaluated, using an in ovo method, the HET-CAM assay, highlighting the biocompatibility and potential anti-irritant effect.

An important factor to consider when evaluating the hepatic effect and toxicity of a compound is the choice of in vitro models that reflect the most relevant liver functions. As for isolated primary human hepatocytes, they rapidly lose their drug metabolism and transport characteristics, which greatly limits their use [30]. An alternative to using primary human hepatocytes is the HepaRG cell line. Analysis of the genetic profile of HepaRG cells showed that they have the characteristics of primary hepatocytes and human liver tissue to a greater extent than other liver cell lines [31].

Thus, for evaluating the effect on cell viability, the hepatocyte cell line—HepaRG—was selected, in which five concentrations of Met (0.5, 1, 1.5, 2 and 2.5 mM) were tested for two time intervals, of 24 and 72 h. Concentrations and their equivalents in mg: 0.173, 0.346, 0.519, 0.692 and 0.865 mg) were selected after extensive evaluation of the literature on in vitro testing of Metformin [32,33,34,35,36]. Additionally, when selecting the tested concentrations, an estimated calculation was made to correlate the in vitro concentrations and the corresponding in vivo doses by applying the formulas described by Levy [37].

Following stimulation for 24 h, an increase in cell viability was observed, at a maximum of approximately 122% in the case of a concentration of 2 mM. In the case of the highest tested concentration, 2.5 mM, cell viability was not significantly affected, with its value being similar to that of unstimulated control cells. After 72 h of stimulation, the effect of Met on cell viability was comparable to that observed after 24 h. In contrast, stimulation with Met for a period of 72 h led to a greater increase in cell viability than 24 h stimulation. Accordingly, the cell viability was approximately 171% at a 2 mM concentration. The highest concentration tested, 2.5 mM, did not influence the cell viability, the value, in this case, being about 108%. In addition, in terms of cell morphology, it did not show any changes, with the cells showing an increased confluence, in accordance with the values obtained from the cell viability test.

To the best of our knowledge, no studies have been performed on the effect of metformin alone on liver cells in vitro. However, Met has been shown to be hepatoprotective in an insulin-resistant and leptin-deficient murine ob/ob model. In a study by Lin and colleagues, it was found that Met can improve fatty liver disease by reversing hepatomegaly, steatosis and aminotransferase abnormalities by inhibiting hepatic expression of tumoral necrosis factor (TNF) and TNF-inducing factors that promote the accumulation of liver lipids [38]. One of the pioneering studies on the effect of metformin on ALT belongs to Marchesini et al. The study found that ALT levels returned to normal after 4 months of metformin treatment in 10 out of 20 patients [13]. Additionally, in a randomized controlled trial led by Jonathan Krakoff, the effect of metformin on alanine aminotransferase (ALT) levels was investigated. The results showed that in the metformin group, the ALT level was slightly lower than in the placebo group [39]. Similar results regarding the effect of metformin on ALT levels were obtained in studies conducted by Uygun [40] and Kadayıfcı [41].

Another study by Conde de la Rosa et al. evaluated the protective effect of Met against apoptosis induced by oxidative stress in primary hepatocytes isolated from rats. The study found that Met protects hepatocytes against oxidative stress-induced caspase activation, poly (ADP-ribose) polymerase (PARP) cleavage and apoptosis [42]. Vilela et al., demonstrated in a murine model that metformin decreases oxidative stress levels and catalase (CAT) and superoxide dismutase (SOD) activity in the gastrocnemius muscle compared to insulin therapy alone, in diabetic rats [43]. Underlying this action of metformin on the SOD and CAT systems may be its ability to block mitochondrial complex I, which leads to the decreased production of superoxide anions in mitochondria [44].

The hepatoprotective effect of Met against drug-induced toxicity has also been evaluated in several in vitro and in vivo studies. Thus, Tripathi et al. evaluated the anti-inflammatory and protective effect of Met against acetaminophen-induced toxicity in the hepatic cell line HepG2. They pointed out that Met inhibits inflammatory responses and reduces liver damage [23]. In vivo, in a murine model, it has been shown that pre-treatment with Met can protect against acetaminophen-induced toxicity by decreasing liver damage and inhibiting acetaminophen-induced prolonged hepatic c-Jun N-terminal kinase (JNK) phosphorylation in wild type mice [45]. Similarly, the potential hepatoprotective effect of Met on the occurrence of carbon tetrachloride-induced liver fibrosis in mice was evaluated. The results showed that treatment with Met suppressed the increase in the liver index, the appearance of fibrosis and the accumulation of connective tissue. These data indicate that Met may have beneficial effects against carbon tetrachloride-induced liver fibrosis [46].

Although Met is widely used in the treatment of type 2 diabetes mellitus, the mechanism of action is not fully known. One of the possible mechanisms underlying the reduction of gluconeogenesis is the inhibition of the mitochondrial respiratory chain complex I or increased cytosolic redox status and decreased mitochondrial redox status due to the inhibition of mitochondrial glycerophosphate dehydrogenase [47]. Starting from the premise that one of the therapeutic targets of Met is mitochondria, in the continuation of the present study, the effect induced by Met on the morphology of the nucleus and mitochondria was evaluated through immunofluorescence studies. The results showed that Met did not induce changes in the structure or number of cellular organs, but at the highest tested concentration of 2.5 mM, a slight condensation of mitochondria was observed. These results are correlated with the results obtained after testing cell viability. Mitochondria play a key role in maintaining the balance between cell survival and death, especially in hepatocytes, by intervening in the intrinsic pathway of apoptosis and in the death of necrotic cells. The main mechanism underlying the cellular transition from survival to cell death is the regulation of membrane permeability [48]. Thus, the size, number and morphology of mitochondria can undergo major changes in the processes of fusion, fission and mitophagy in the presence of harmful stimuli [49]. In addition, in terms of morphology, the structure and number of nuclei are not affected by Met treatment. None of the five concentrations tested cause nuclear condensation, a sign of cellular apoptosis. These results support the hypothesis that Met has no cytotoxic effect on healthy hepatocytes.

Cell migration is an important step in wound healing [50]. In the process of liver regeneration, activated hepatic stellate cells are involved in the production of extracellular matrix, including collagen to fix the architecture of the lesion texture, a process similar to the wound healing process. Thus, the extracellular matrix serves as a support for hepatocyte proliferation and maintains mechanical stability in the injured area [51]. Due to these considerations, the present study evaluated the effect of Met on cell migration by the scratch assay. The results showed that Met increases the dose-dependent cell migration of hepatocytes, which may explain its potential protective and repairing effect on liver damage. In addition, it is known that patients with type 2 diabetes mellitus are affected by changes in the ability to heal wounds. Thus, the potential repair effect of Met was evaluated in a murine model, noting that treatment with Met may accelerate wound healing [52]. Currently, the role played by Met in the function of endothelial precursor cells and in wound healing is intensely disputed. Previous studies have shown that Met accelerates wound healing and increases the amount of circulating endothelial precursor cells [53,54]. However, another study has argued the opposite and even shown that Met delays wound healing [55]. The results of the present study showed that in vitro, Met stimulates cell proliferation and migration, key factors in the repair process.

For a more general picture of the effect induced by Met, the present study evaluated its action in blood vessels, both in terms of biocompatibility and anti-irritant potential, by using the chorioallantoic membrane of the hen’s egg. The obtained results showed that Met does not induce irritating effects at the level of the vascular plexus, having an irritation score of 0.7, which indicates that it is a substance without toxic effects. In addition, with respect to its potential protective role, pre-treatment with Met significantly decreased the irritant effects induced by SDS on the capillaries. Thus, if in the absence of pretreatment, the irritation score induced by SDS was 19.68, in the case of pre-treatment, it decreased to 7.29. These results support previous findings that Met increases cell viability and stimulates cell migration with a potential impact on wound healing. As far as we know, Met has not been previously tested for irritant and anti-irritant effects by the HET-CAM method. However, Met has previously been applied to the chorioallantoic membrane to assess its potential effect on angiogenesis. Thus, Wang and colleagues used the CAM method to highlight the antiangiogenic effect of Met in combination with Pemetrexed [56]. Novel aspects of this study include the choice of a broader range of test concentrations and evaluation times. In the majority of in vitro studies investigating the therapeutic effects of metformin, a single or a few concentrations were chosen. As far as we are aware, the effects of metformin on the hepatocyte cell line—HepaRG—have not been evaluated in terms of both morphology and migration rate. An additional novelty of this study is the in ovo evaluation of metformin. The anti-irritant effect of metformin has not been evaluated on the chorioallantoic membrane to date. All of these studies and preliminary results support metformin’s hepatoprotective properties.

## 5. Conclusions

Metformin has been used in clinical practice for over 50 years and is considered the therapy of choice for the treatment of type 2 diabetes. However, the effects of Met are not limited to the hypoglycemic effect. The results of the present study indicate that Met does not induce toxic effects in hepatocytes, but on the contrary, causes a stimulation of cell proliferation. In addition, by evaluating cell morphology and observing the structure, organization, and number of mitochondria and nuclei, it was confirmed that Met does not induce alterations in their characteristics. On the contrary, by applying the Scratch technique, it was found that Met stimulates cell migration, with a possibly beneficial effect on wound healing. Ovo studies complemented in vitro studies, supporting that Met is free of toxic and irritating effects, and has anti-inflammatory and protective effects against vascular irritation. In conclusion, Met presents a safety profile at the level of hepatocytes with possible hepatoprotective actions. However, further studies are needed to determine the possible biological mechanisms underlying this therapeutic action.

## Figures and Tables

**Table 1:** Irritation score values for the positive control (SDS 1%), negative control (distilled water) and Met 2 mM.

	H_2_O	SDS 1%	Met 2 mM
*IS*	0.13	19.68	0.7
tH	300	22 s	300 s
tL	300	19 s	300 s
tC	298	17 s	279 s

**Table 2:** Anti-irritant effects of Met at a concentration of 2 mM and solvent H_2_O after CAM irritation with SDS 1%.

	SDS 1%	H_2_O + SDS 1%	Met 2 mM + SDS 1%
*IS*	19.68	17.58	7.29
tH	22 s	78 s	270 s
tL	19 s	55 s	197 s
tC	17 s	30 s	156 s
H_AI_		3.54	12.27
L_AI_		2.89	10.36
C_AI_		1.76	9.17

## Data Availability

Not applicable.
